# Treating the perimenopause in the UK Armed Forces: a mixed-methods review exploring the confidence of GPs

**DOI:** 10.3399/BJGPO.2024.0088

**Published:** 2025-02-26

**Authors:** Antony Sean Willman, Kate King

**Affiliations:** 1 College of Medical and Dental Sciences, University of Birmingham, Birmingham, UK; 2 Queen Elizabeth Memorial Health Centre, Defence Primary Healthcare, Wiltshire, UK; 3 Academic Department of Military General Practice & Primary Care, Research & Clinical Innovation, ICT Centre, Birmingham, UK

**Keywords:** perimenopause, defence, women's health, general practitioners, primary healthcare

## Abstract

**Background:**

Access to high quality perimenopause (PMP) care for UK Armed Forces (UKAF) personnel is crucial, given the increasing proportion of women aged 40–55 in the service. However, owing to the lack of exposure of GPs to the PMP in defence primary health care (DPHC), there are concerns about the confidence in PMP management, particularly in prescribing hormone replacement therapy (HRT).

**Aim:**

To assess the confidence of GPs working in DPHC in the management of the PMP.

**Design & setting:**

This study employed a mixed-methods approach and included all GPs (uniformed and civilian) working in DPHC.

**Method:**

A cross-sectional survey gathered quantitative data on demographics, views on PMP care, and self-rated confidence levels in managing the PMP among defence GPs (DGPs). Semi-structured interviews of purposefully sampled responders were thematically analysed to explore these issues further.

**Results:**

There were 164 responses from 542 DGPs (response rate 30.3%). The majority of responders expressed confidence in managing the PMP but reported lower confidence levels in prescribing HRT for younger women and initiating testosterone. Factors influencing confidence included recent PMP continuing professional development (CPD), GP sex, and exposure to PMP cases. Semi-structured interviews provided deeper insights into GP characteristics, CPD, and awareness of the PMP. Women’s health hubs providing PMP care and experiential education were strongly supported.

**Conclusion:**

The study identified gaps in confidence among DGPs, particularly in certain aspects of PMP management, similar to those found in NHS GPs. CPD and case exposure were important predictors of confidence, with strong support for regional women’s health hubs to optimise PMP care. Further research is warranted to explore strategies for bridging confidence gaps and improving PMP care delivery within the UKAF context.

## How this fits in

There is currently a paucity of published literature on the primary care management of the perimenopause within the UK Armed Forces. This study demonstrated self-rated confidence in perimenopause (PMP) management was positively affected by female sex, exposure to PMP cases, and undertaking education in women’s health and managing the PMP. Being a uniformed GP (UGP) was not significant but the lower response rate, especially from males, may have biased this finding. Thematic analysis of interviews identified GP characteristics (including sex and role), continuing professional development (especially experiential), and PMP awareness. The introduction of women’s health hubs was identified as a way to provide and improve PMP care, as well as an educational opportunity for GPs wishing to gain experiential learning through exposure to cases.

## Introduction

Reliable access to high quality PMP care for UK Armed Forces (UKAF) personnel was found to be a prevalent theme in a study of personnel serving through the PMP.^1^ This survey of women in the UKAF with self-reported PMP symptoms found that 54.4% of responders felt their treatment could be improved. They reported work-related stress and common mental health disorders being diagnosed instead of the PMP, and several reported the GP had a lack of confidence in PMP management. The proportion of potentially perimenopausal women serving in the UKAF (aged 40–60 years) rose from 11%, in 2012 to 19% in 2024,^2^ meaning that good quality PMP care needs to be available and accessible for serving personnel.

In the wider UK population, the number of women between the ages of 50 and 64 years increased from 5.1 million in 2011 to 5.9 million in 2021,^3^ and more women are being prescribed hormone replacement therapy (HRT) for PMP symptoms.^4^ Despite this, GP confidence in HRT prescribing for the PMP is lacking, especially in women aged <45 years and those presenting with predominantly psychological symptoms.^5,6^ In one survey assessing the potential demand for a specialist PMP service, more than 50% of NHS GPs reported seeing >4 PMP cases a week yet still lacked confidence.^7^

While the numbers of potentially perimenopausal women in the UKAF are increasing, women are still a small proportion of the overall UKAF population (11.8%).^2^ Defence primary health care (DPHC) provides occupationally focused primary health care for entitled UKAF personnel. There is a higher prevalence of musculoskeletal injuries and minor mental health disorders than in the civilian sector with typically fewer long-term conditions.^8^ GPs working in DPHC are either civilian defence employed (defence GPs; DGPs) or serving uniformed GPs (UGPs). DGPs tend to be static, providing continuity of care. UGPs are more likely to be working in front-line deployable units and have multiple roles supporting their unit in addition to providing clinical care. ^9^ The rising numbers of women aged 40–60 years are more likely to be more senior in rank and so concentrated in rear echelon and headquarters roles, away from front-line units. For many GPs in the UKAF, regardless of continuing professional development (CPD) undertaken in women’s health and the PMP, exposure to just a handful of PMP cases a year may result in low or non-existent confidence levels.^10^ This study therefore aimed to better understand themes identified in our previous work by exploring DGPs’ confidence in managing the PMP.^3–6,11,12^

## Method

This study used a sequential, explanatory mixed-method design. Phase 1 was a cross-sectional survey used to gather quantitative data. The results of this informed and linked to phase 2, semi-structured interviews of purposefully sampled survey responders to gather qualitative data and expand on phase 1. The project was undertaken in DPHC with both DGP and UGP participants.

### Quantitative data

A questionnaire was constructed to gather data on demographics (GP role, sex, CPD, practice demographics, and practice population), views on PMP care in DPHC with 10 agree or disagree Likert-type questions, and self-rating questions on confidence in managing the PMP. This comprised of 10 statements based on themes encountered regularly in DPHC, and on previous research in this area.^7,13–16^ These were 4-point confident or not confident Likert-type questions that when summed, gave a total confidence score (TCS) of 0–30. A REDCap survey was constructed using the questions and distributed to all GPs in defence via official email and unofficial social networking channels.^17^ Several requests to complete the survey were sent out using the same routes, but reminders were not sent to participants to promote completion. Survey results were imported into Microsoft Excel to calculate descriptive statistics. To explore demographic factors that might explain the TCS, a multivariable linear regression model was calculated using Stata (version 18).^18^ Responders were invited to leave an email address if they were happy to be interviewed.

### Qualitative data

A purposeful sample of 11 responders covering sex and role was invited for interview, using the email addresses supplied in the survey. Semi-structured interviews of 15–40 minutes were undertaken in person and remotely. Recordings were uploaded to Otter.ai for automated transcription,^19^ with the transcripts then corrected and imported into NVivo (R1 for MacOS) for analysis.^20^ An inductive iterative process identified codes, concepts, and themes from the free text. Constant comparison between the researchers ensured mutually agreed concordant themes.

## Results

### Quantitative data: survey

The overall response rate for GPs completing the survey was 30.3% (*n* = 164/542). Table 1 gives a breakdown of completed surveys by role and sex of GP. The response rate was highest for female DGPs (46.1%, *n* = 77/167) and lowest for male UGPs (15.5%, *n* = 32/206). There was a survey non-completion rate of 28.4% (164 completed from 229 started).

**Table 1.** Breakdown of survey responders by role and sex

Characteristic	Frequency, *n* (%)	As proportion of total DPHC GP population, % (denominator)
Role		
Defence GP	102 (62.2)	43.2 (236)
Uniformed GP	56 (34.1)	18.3 (306)
Rather not say	5 (3)	
Missing	1 (0.6)	
Sex		
Female	100 (61.0)	37.5 (267)
Male	59 (36.0)	21.5 (275)
Rather not say	3 (1.8)	
Missing	2 (1.2)	

DPHC = defence primary health care.

DGPs were more experienced than UGPs with 39.2% having more than 20 years since completion of training as a GP compared with 14.3%. DGPs had more PMP case exposure: 19.6% had seen more than 20 cases in the previous 12 months compared with 3.6% of UGPs, and 82.2% UGPs had ≤10 PMP consults in the previous 12 months compared with 54.9% DGPs. Compared with UGPs, DGPs were slightly more likely to have done women’s health CPD (87.3% versus 71.4%) and PMP and HRT CPD (82.4% versus 60.7%) in the previous 24 months. The practice populations were positively skewed ranging from 400–14 000 with a median value of 2500 (interquartile range 1500–4000). A full breakdown of responses is shown in Supplementary Table 1.

The majority (*n* = 144, 87.8%) agreed or strongly agreed with the statement ‘the PMP can often present with multiple symptoms so can be difficult to diagnose’. Only 12.8% (*n* = 21) agreed that GPs in DPHC had adequate training in PMP management and 76.8% (*n* = 126) agreed that ‘regular and frequent experience in managing women with PMP symptoms is important for a successful outcome'. A full breakdown of responses is shown in Supplementary Table 2.

Of the self-rating Likert-type questions, just over half (*n* = 89, 54.3%) were confident or very confident in managing the PMP, with a similar figure for initiating HRT for psychological symptoms of the PMP (*n* = 85, 51.8%). Most (*n* = 119, 72.6%) were confident or very confident in continuing HRT prescribing for the PMP. Fewer responders were confident or very confident in initiating HRT for women aged <40 years (*n* = 46, 28.1%), initiating HRT based on a PMP rating scale (*n* = 54, 32.9%), and initiating testosterone for PMP symptoms (*n* = 14, 8.5%). A full breakdown of responses is shown in Supplementary Table 3.

The calculated TCS had a normal distribution with a mean of 13.64 (95% confidence interval [CI] = 12.61 to 14.67). The internal reliability of the TCS had a calculated Cronbach’s alpha of 0.93. Results of the multivariable linear regression model are presented in Table 2. The major explanatory variable for TCS was number of PMP cases seen with all positively affecting scores (*P*<0.001). Sex (coefficient 2.17, 95% CI = 0.26 to 4.08, *P*<0.05) and HRT CPD (coefficient 2.78, 95% CI = 0.31 to 5.25, *P*<0.05) also had a positive effect.

**Table 2.** Multivariable linear regression model: TCS (0–30) as dependent outcome variable. Effect of explanatory variables

Explanatory variable	Coefficient	SE	95% CI	*T*-statistic	*P* value
Families practice?					
No	Base				
Yes	-0.495	0.878	-2.231 to 1.241	-0.563	0.574
Approximate number of PMP consults in the past 12 months?					
*None*	Base				
1–10	4.20	1.32	1.59 to 6.81	3.18	0.002*
11–20	9.16	1.65	5.89 to 12.43	5.542	<0.001*
21–30	8.52	1.93	4.71 to 12.34	4.42	<0.001*
>30	14.80	2.23	10.39 to 19.22	6.63	<0.001*
Recruits in practice?					
No	Base				
Yes	1.09	0.86	-0.61 to 2.79	1.26	0.208
Years since CCT					
≤5 years	Base				
6–10 years	-0.55	1.31	-3.13 to 2.04	-0.42	0.68
11–15 years	-0.25	1.51	-3.24 to 2.74	-0.16	0.87
16–20 years	0.04	1.52	-2.97 to 3.04	0.02	0.98
>20 years	1.72	1.33	-0.92 to 4.35	1.29	0.20
CPD in any women’s health subject area in past 24 months?					
No	Base				
Yes	0.51	1.39	-2.24 to 3.26	0.37	0.72
CPD in PMP and HRT in past 24 months?					
No	Base				
Yes	2.78	1.25	0.31 to 5.25	2.22	0.03*
What is your sex?					
Male	Base				
Female	2.17	0.97	0.26 to 4.08	2.24	0.03*
What is your role?					
DGP	Base				
UGP	1.79	1.11	-0.41 to 3.98	1.61	0.11
Constant	3.29	2.10	-0.85 - 7.44	1.57	0.12

CCT = Certificate of Completion of Training. CPD = continuing professional development. DGP = defence GP. HRT = hormone replacement therapy. PMP = perimenopause. SE = standard error. TCS = total confidence score. UGP = uniformed GP.

Bold and asterisked = statistically significant.

### Qualitative data: Thematic analysis of interviews

The following three themes were identified relating to confidence in managing the PMP: GP characteristics; CPD; and awareness of the PMP. For the excerpts, UGP is uniformed GP, DGP is defence GP, F is female, M is male.

#### GP characteristics

These related mainly to sex and role.

##### Sex

Women may prefer seeing a female GP,^21^ and this would lead to increased exposure to the PMP, improving confidence. Although there was general agreement, there was also some dissonance. An alternative view was that female GPs might be less sympathetic, that women experiencing the PMP should simply 'power through' as it was a stage of life. Others felt male GPs, although less confident, might be more thorough for fear of missing something. Some interviewees suggested that male GPs lacking in confidence would refer patients with PMP to another GP, usually female, who was more likely to have a women’s health interest:

*‘Unless they have that interest in women’s health, they're not focusing on it. And so unless you have a particular interest … it’s almost become easier for colleagues to just refer to another colleague, or say, or why don't you go and see that person, they have an interest in women’s health.’* (UGP-F5)

##### Role

Among UGPs, interviewees universally recognised their limited PMP case exposure and time demands meant their confidence in PMP management was lacking. This did not mean they did not want to help their patients, rather the limited time they had in clinical practice was focused on their younger, predominantly male, demographic:

*‘So, in terms of having time available to perhaps identify areas of knowledge weakness, or your own PUNS* [Patient’s Unmet Needs] *and DENS* [Doctor’s Educational Needs]*, you don't have the time for that. In addition, you're then deployed, … exercises, … operations, … you might have the odd patient that might present but it’s going to be rare.’* (UGP-F6)

UGPs spoke about prioritising their CPD to those clinical areas they were most exposed to, and stated they had undertaken less PMP and HRT CPD compared with DGPs. There was agreement among DGPs that this was to be expected of their uniformed colleagues, and that this would lead to reduced confidence in managing the PMP.

### Continuing professional development

CPD was a common theme among interviewees, both prompted and unprompted.

#### Experiential learning

There was a complex interplay between experiential learning, sex dynamics, professional engagement, and contextual factors that shaped a GP's confidence in managing the PMP. For example, the engagement of GPs in CPD, their interest in women’s health, and practising it in primary care emerged as influential factors. GPs who perceived women’s health as an integral part of their professional role exhibited greater interest and willingness to develop their skills in PMP management, thereby improving their confidence. There was almost universal support for regional women’s health hubs throughout DPHC to focus provision of clinical management advice as well as opportunities for experiential learning. It was felt this would be a key enabler to improving confidence:

*‘I think the main one probably is the lack of patients. I think it really boils down to how many patients you're seeing. And if you're not seeing very many, you de-skill, and then it’s much more difficult to look after those patients.’* (DGP-F1)

#### Learning style

Mixed views were expressed by different interviewees about PMP-specific training. Some felt that the menopause being part of the GP curriculum was improving confidence. The style and type of learning was also mentioned by interviewees. Most preferred small group learning where challenges and lack of confidence were shared, but others found the convenience of short webinars, podcasts, and didactic learning events more to their liking. Responders did not reflect on the effectiveness of different learning types for long-term knowledge retention.

### Awareness of the PMP

Almost all interviewees described the PMP as a hot topic, with demand for HRT being driven by celebrity endorsement and a strong media presence.^22^

#### Patient demand

The rising awareness of the PMP, the role of HRT, and demand from patients had led to self-directed learning. GPs who had done some focused learning were more confident to undertake opportunistic inquiry about PMP symptoms, while recognising presentation can be very varied and nuanced. Some interviewees spoke about the expert-informed patient instilling fear into some GPs; the so called 'Davina effect’ (mentioned by four interviewees). For some, this prompted engaging with PMP CPD, which in turn, improved confidence in managing these consultations:

*'And I think the other the other point is that potentially perimenopausal patients can present with a cluster of symptoms … And as a GP, that can feel a bit overwhelming when they say, "well, my mood is low, but I've also got, you know, dryness, I've also got skin issues."’* (UGP M1)

#### COVID-19 and remote consulting

Some felt the COVID-19 pandemic had driven demand, with easier access to GPs via remote consulting systems as a possible factor. This view is echoed in studies looking at the unintended consequences of online consultations.^23,24^ Interviewees stated this increase in demand had made GPs more aware of the PMP, the multitude of symptoms, and the role of HRT, and therefore they felt more confident to manage it.

## Discussion

### Summary

Overall, just over half of responders felt confident in managing the PMP; only slightly lower than one recent survey of NHS GPs (54.3% versus 60.7%).^16^ There was reduced confidence prescribing HRT to younger women aged <40 years (28.1%) and in the use of testosterone (8.5%), these results are less than those found in another survey of primary care practitioners (77% GPs) where the figures were 44% and 29%, respectively.^6^ Recent PMP CPD, GP sex, and PMP case exposure were the major factors affecting clinician confidence in managing the PMP. These findings are likely to be interlinked with no one predominant driving factor leading to improved confidence. Female GPs appear more likely to have an interest in women’s health, this will in turn drive their CPD choices.^25^ Additionally, women with PMP symptoms may preferentially seek a female GP assuming better knowledge and a more understanding ear.^22^ This in turn leads to further case exposure and improved confidence with a corresponding fall in exposure, skill loss, and reducing confidence levels in male GPs. Interviewees recognised the importance of CPD and PMP awareness (including as part of the Royal College of General Practitioners [RCGP] curriculum). Small group learning was mentioned and has proven popular in DPHC, so may have played a part.^26^ UGPs were more recently qualified, and complex PMP cases, while challenging for GP registrars, can promote learning.^27^ The interaction between this residual PMP knowledge, awareness of the PMP and HRT, and re-exposure to just a few cases may have resulted schema retrieval and improved confidence.^28^ Further research may be needed to confirm this.

### Strengths and limitations

A major limitation of this study is the suggested high non-completion rate for the survey (28.4%). Analysis of participants showed that all personnel consenting to take part completed the survey but 65 (of 229 total participants, 28.4%) did not complete consent. Owing to the anonymous nature of the survey, inferences cannot be drawn as to the reasons for this non-completion, or the demographics of those opting not to participate. Additionally, the option on the survey to save and complete later was not enabled, which could have contributed to this non-completion rate.

Being a UGP was a factor affecting confidence but not significantly, which was unexpected given their clinical exposure is most limited of all DGP groups. This group were a younger demographic and so closer to their GP licensing exams, but there may also be a selection bias, with the low response rate (18.3%) impacting this finding. We hypothesise that those lacking confidence in PMP care were less likely to respond, meaning the confidence of male UGPs may be worse than is represented here. As the interviewees were selected from this cohort, there is a risk that not all qualitative themes were teased out.

### Comparison with existing literature

Levels of PMP-specific training over the previous 2 years were high (*n* = 122, 74.8%), but case exposure low. Interviewees talked about ‘training we can get from those that do it in real life’ (4 participants) and being ‘a fan of practical face-to-face learning’ (4 participants). This experience is a fundamental facet of adult learning.^29^ Christianson *et al* found similar when assessing the menopause learning needs of resident doctors, and Hess *et al* found regular contact with PMP women improved knowledge scores in line with guidelines.^30,31^ This is relevant when considering the role of women’s health hubs and their educational role, as well as service delivery.

While this study explored the confidence of DGPs, its findings align with the literature on UK civilian GPs.^7^ The improved confidence that comes from focused CPD, case exposure, and discussion in defence is generalisable to the NHS, with similar positive learning outcomes in initiatives such as practice-based small group learning.^25,32^ It is interesting to note that since the data collection for the study exploring patients’ experiences with PMP in defence, the majority of responders have undertaken some form of CPD on the topic.^1^ This suggests a GP cadre recognition that knowledge needed to be improved in line with the patient view at the time. A repeat of the patient experience study may now reveal different results.

### Implications for practice

The study reinforces the need for practical, experiential training opportunities. High levels of PMP-specific training were reported, yet actual case exposure was low, which in turn affected confidence. While the relationship between confidence and competence is complex, for defence, both could be assessed and self-efficacy improved by case exposure in specific women’s health clinics, a view supported by most responders and interviewees.^26^ Women’s health hubs are being evaluated as part of the NHS women’s health strategy, and early indications are of positive cost versus benefit ratio, with improved access to women’s health services, including PMP care.^27,28^

In the UKAF, specifically, the small female patient population in many locations further exacerbates the risk of unequal access and quality of care. The introduction of services equivalent to NHS women’s health hubs could overcome this for the UKAF. This study suggests their development would be positively received by DGPs, allowing the delivery of PMP care by upskilled GPs, peripatetic specialists, and a focused learning environment for DGPs. However, their implementation must be evaluated, similarly to that being undertaken by the Birmingham, RAND, and Cambridge Evaluation (BRACE) Centre, in order to demonstrate that these effects are realised.^29^ The UKAF have the ability to enable GP placements in clinical environments (such as hubs) to improve their PMP and general women’s health knowledge. This could mitigatate the risk of de-skilling, as well as identifying common knowledge gaps.

The PMP remains a hot topic in the media and there had been concerns that DGPs were not able to provide adequate clinical care to those serving through this important period of a woman’s life. This study shows that, while there are gaps and groups lacking in confidence, DGPs are, overall, akin to their civilian counterparts in their knowledge and confidence in this area of care.

## Data

The dataset relied on in this article is available from the corresponding author on reasonable request.
